# Natural Hirudin Increases Rat Flap Viability by Anti-Inflammation via PARs/p38/NF-*κ*B Pathway

**DOI:** 10.1155/2015/597264

**Published:** 2015-12-07

**Authors:** Liu Peng, Xinyuan Pan, Guoqian Yin

**Affiliations:** ^1^Guangxi Medical University, Nanning, Guangxi 530021, China; ^2^Department of Plastic and Aesthetic Surgery, The First Affiliated Hospital of Guangxi Medical University, Nanning, Guangxi 530021, China

## Abstract

The present study aimed to evaluate the effect of natural hirudin on rat random skin flap viability and to determine the mechanism. Forty-eight rats were randomly divided into 2 groups. After the dorsal skin flap operation (3 cm × 10 cm in size), subcutaneous injections of 6 ATU hirudin were administered to group H (*n* = 24) every 12 h, while group C (*n* = 24) received an equal volume of 0.9% normal saline. Six rats from each group were euthanized 1, 2, 4, and 7 days after the operation. A full skin sample was collected from these rats to measure the p38-mitogen-activated protein kinase (p38-MAPK), phospho-p38- (Pp38-) MAPK, nuclear factor-*κ*B (NF-*κ*B) p65, phosphor-NF-*κ*B (pNF-*κ*B) p65, tumour necrosis factor- (TNF-) *α*, interleukin- (IL-) 6, and intercellular adhesion molecule- (ICAM-) 1 levels via western blot (WB) assays. The results showed that flap viability was significantly higher in the hirudin-treated group, which showed a reduced inflammatory response compared with the control group. The Pp38/p38, pNF-*κ*B p65/NF-*κ*B p65, TNF-*α*, IL-6, and ICAM-1 levels in the hirudin-treated group were lower than those in the control group. The results demonstrated that hirudin could improve random skin flap viability and suggested that this effect maybe occurs by blocking the thrombin/proteinase-activated receptors (PARs)/p38/NF-*κ*B signalling pathway, thus decreasing the inflammatory response.

## 1. Introduction

Random pattern skin flaps are commonly used to repair skin defects during plastic and reconstructive surgery, although flap necrosis remains a problem. Many medications, such as sympatholytics, vasodilators, calcium channel blockers, prostaglandin inhibitors, glucocorticoids, and free radical scavengers, have been reported to be effective for increasing flap viability [1, 2]. However, these drugs cannot entirely prevent flap necrosis. Additionally, many side effects could occur or are associated with systemic application of these drugs at high doses, which are often required to produce satisfactory effects.

Some studies have shown that natural hirudin can increase flap viability in animal experiments. The possible mechanism may involve improving microcirculation and antihyperoxidation. However, whether hirudin can influence inflammation following ischaemia and congestion is still unknown. The aim of the present study was to evaluate the effect of hirudin on inflammation in rat random pattern skin flaps and to explore its underlying mechanism.

## 2. Materials and Methods

### 2.1. Animals and Reagents

Forty-eight male Sprague-Dawley (SD) rats weighing between 250 and 300 grams were obtained from the Animal Experimental Centre of Guangxi Medical University. The animal handling protocol was in accordance with the Regulations of Laboratory Animal Care [3]. The rats were housed singly in polycarbonate cages at 24°C in a humidity-controlled environment with a 12-hour day/night cycle during the preoperative and postoperative periods. The rats were allowed free access to water and rat chow.

Lyophilized natural hirudin powder (Patent number ZL03113566.8, Lot number KK-001) was provided by Nanning JinXueHuang Bioengineering Co. Ltd. (Guangxi, China). Anti-Pp38-MAPK rabbit antibody (cat. #4511), anti-p38-MAPK rabbit antibody (cat. #8690), and anti-GAPDH rabbit antibody (cat. #5174) were purchased from Cell Signalling Technology (Beverly, MA, USA). Anti-ICAM-1 mouse antibody (sc-8439), anti-TNF-*α* rabbit antibody (sc-8301), anti-IL-6 rabbit antibody (sc-1265-R), anti-NF-*κ*B p65 rabbit antibody (sc-372), anti-pNF-*κ*B rabbit antibody (sc-101748), and anti-GAPDH mouse antibody (sc-365062) were purchased from Santa Cruz Biotechnology (Santa Cruz, CA, USA). The secondary antibodies (anti-rabbit IRDye-800CW and anti-mouse IRDye-800CW) were purchased from LI-COR Biosciences (Lincoln, NE, USA).

### 2.2. Surgical Procedures and Flap Evaluation

**Figure 1:**
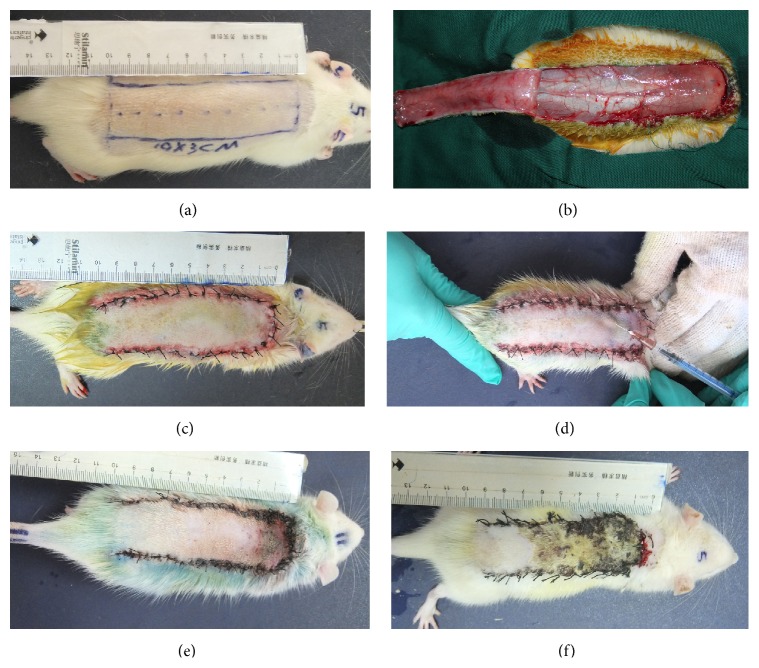
Images of the surgical procedures and flap evaluation. (a) A 3 cm × 9 cm flap was designed. (b) The flap was elevated. (c) The skin flaps were resutured to their original bed with 4-0 nylon sutures. (d) Injection of hirudin or 0.9% normal saline was performed. (e) A skin flap from group H on postoperative day 7 is shown. (f) A skin flap from group C on postoperative day 7 is shown.

All of the rats were fixed on a board after being anesthetized with intraperitoneal injections of 10% chloral hydrate at a dose of 3 mL/kg body weight. The dorsal trunk skin was shaved with electric clippers and prepared with Betadine and alcohol. A 3 cm × 10 cm flap was designed with palpable hip joints as anatomical landmarks for the base. Under sterile conditions, incisions were made, and the entire flap was cut below the level of the panniculus carnosus [4]. The flap was elevated and inspected for direct axial or muscular perforating cutaneous blood vessels supplying the base. These vessels, if found, were cauterized to ensure a random skin flap. After obtaining a 0.3 cm × 3 cm full skin sample from the distal edge on day 0, the skin flaps were resutured to their original beds with 4-0 nylon sutures (Figures 1(a)–1(c)).

The rats were then randomly divided into two groups according to the treatment they received. In group H (*n* = 24), six symmetrical points from 3 cm to 6 cm distal to the skin flap base were selected, and 1 ATU hirudin was administered at each point by subcutaneous injection every 12 h until the animals were sacrificed. Group C (*n* = 24) served as the control group and received an equal volume of the vehicle (0.9% normal saline) (Figure 1(d)).

Six rats from each group were anaesthetized, and the flattened back skin flaps were photographed with a digital camera before they were sacrificed at 1, 2, 4, and 7 days after the operation (Figures 1(e)–1(f)). The pictures were analysed with Image-Pro 6.0 software (TIANGE, Ltd, USA) to calculate the total area, survival area, necrosis area, and flap survival rate:

(1) $$\begin{array}{c} Flap\;\;survival\;\;rate=\left(\;\frac{flap\;\;survival\;\;area}{total\;\;area}\;\right)\times 100\%. \end{array}$$

Flap necrosis was defined by a dark colour and eschar formation.

### 2.3. Measurement of IL-6, TNF-*α*, ICAM-1, p38, Pp38, NF-*κ*B, and pNF-*κ*B Levels

At postoperative days 1, 2, 4, and 7, six randomly chosen rats from each group were sacrificed after the skin flaps were photographed. A 1 cm × 4 cm full skin sample was collected from the middle of the flap to semiquantitatively measure the Pp38, p38, pNF-*κ*B p65, NF-*κ*B p65, TNF-*α*, IL-6, and ICAM-1 levels by western blot (WB) assay.

The skin tissues were homogenized in RIPA buffer (ProMab Biotechnologies, USA) containing 0.5% Nonidet P-40, 10 mM Tris, pH7.4, 150 mM NaCl, 1 mM ethylenediaminetetraacetic acid (EDTA), 1 mM Na_3_VO_4_, 1 mM phenylmethylsulfonyl fluoride (PMSF), and phosphatase inhibitors (PhosSTOP, Roche, Mannheim, Germany) for 30 min, and then the homogenate was centrifuged at 14,000 g for 5 min. Then, proteins (50 *μ*g) from the supernatant of each sample were separated by SDS-PAGE and transferred onto polyvinylidene difluoride membranes (Millipore, Bedford, MA, USA), which were subsequently blocked with 3% BSA/TBS-T for 1 h at room temperature. The membranes were incubated with appropriate primary antibody dilutions overnight at 4°C. The membranes were washed twice for 10 min with 0.1% Tween phosphate buffer solution (PBST) and then incubated with goat anti-rabbit IR-Dye 800CW-labeled secondary antibody (1 : 5000, Li-Cor, Lincoln, NE, USA) for 2 h at room temperature. After two 10 min washes in PBST, the membranes were imaged using a Li-Cor Odyssey scanner (Li-Cor, Lincoln, NE, USA). Boxes were manually placed around each band of interest, which were used to calculate the near-infrared fluorescent raw intensity values with the intralane background subtracted using the Odyssey 3.0 analytical software (Li-Cor, Lincoln, NE, USA).

The near-infrared fluorescence values for TNF-*α*, IL-6, and ICAM-1 were normalized to that of GAPDH. The ratios of Pp38 to p38 and pNF-*κ*B to NF-*κ*B were also calculated.

### 2.4. Statistical Analyses

All of the results were expressed as means ± standard deviation (SD). Statistical analysis of the data between groups was performed using independent samples *t*-tests, and analysis of variance was used to analyse the data within each group. Significance was set at *P* < 0.05. Analyses were performed using the Statistical Package for Social Sciences (SPSS) for Windows version 16.0 (SPSS Inc., Chicago, IL, USA).

## 3. Results

### 3.1. Flap Survival

Necrosis became evident between the second and fourth days, starting at the distal part of the flap, and was well demarcated by the end of one week (Figures 1(d) and 1(e)). The flap survival rate in group H was higher than that in group C (78.54 ± 2.8% versus 64.62 ± 3.20, resp., *P* < 0.05).

### 3.2. TNF-*α*, IL-6, and ICAM-1 Levels in the Flaps

**Figure 2:**
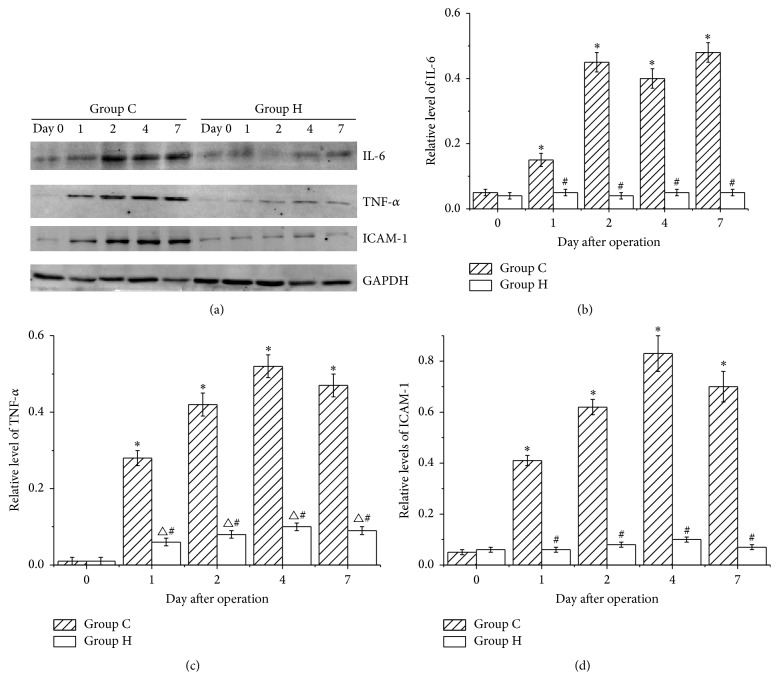
TNF-*α*, IL-6, and ICAM-1 levels in the skin flaps. (a) WB images of TNF-*α*, IL-6, ICAM-1, and GAPDH. (b) IL-6 levels in groups C and H. The IL-6 levels in group C were significantly increased after the operation (^*∗*^ *P* < 0.05 versus day 0). The IL-6 levels in group H demonstrated no significant changes and were lower than those in group C (^#^ *P* < 0.05 versus group C). (c) TNF-*α* levels in groups C and H. The TNF-*α* levels in the two groups were significantly increased after the operation (^*∗*^ *P* < 0.05 versus day 0, ^△^ *P* < 0.05 versus day 0). The TNF-*α* levels in group H were lower than those in group C (^#^ *P* < 0.05 versus group C). (d) ICAM-1 levels in groups C and H. The ICAM-1 levels in group C were significantly increased after the operation (^*∗*^ *P* < 0.05 versus day 0). The ICAM-1 levels in group H were increased after the operation, but no significant differences were observed between the days, and the levels were lower than those in group C (^#^ *P* < 0.05 versus group C).

WB images of TNF-*α*, IL-6, ICAM-1, and GAPDH are shown in Figure 2(a). The IL-6 levels in the skin flaps of group C were significantly increased from postoperative day 2 to day 7 (*P* < 0.05 versus day 0). In contrast, there were no significant changes in the IL-6 levels of group H, which were lower than those of group C (*P* < 0.05 versus group C) (Figure 2(b)).

After the operation, the TNF-*α* levels in the skin flap in group C were significantly increased (*P* < 0.05 versus day 0), peaking on postoperative day 4. The TNF-*α* levels were also significantly increased in group H (*P* < 0.05 versus day 0), although the levels were lower than those of group C (*P* < 0.05 versus group C) (Figure 2(c)).

The ICAM-1 level trend in group C was similar to that of TNF-*α*. However, no significant increases in ICAM-1 levels were observed in group H, and the levels were lower than those in group C (*P* < 0.05 versus group C) (Figure 2(d)).

### 3.3. Pp38/p38 Levels in the Flaps

**Figure 3:**
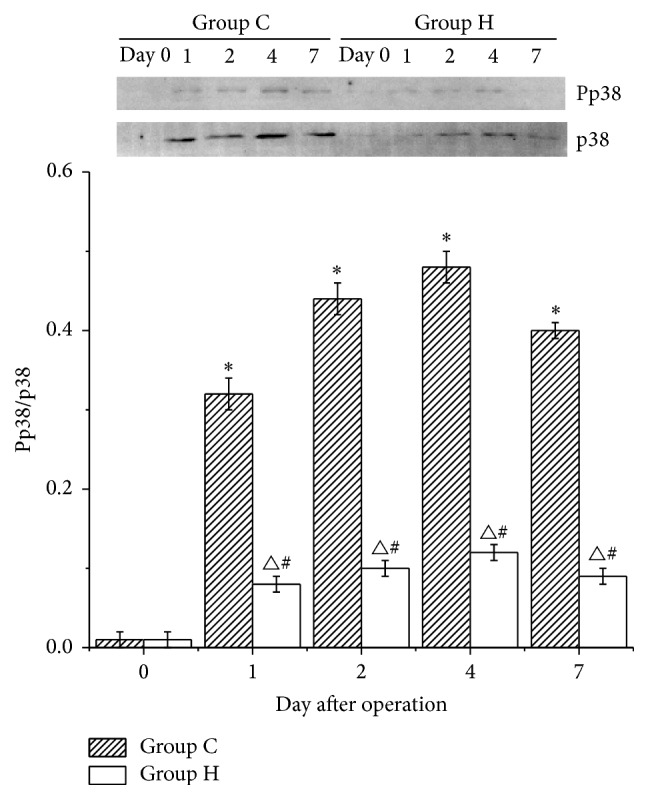
Pp38/p38 levels in groups C and H. The Pp38/p38 levels in groups C and H were significantly increased after the operation (^*∗*^ *P* < 0.05 versus day 0, ^△^ *P* < 0.05 versus day 0) and peaked on postoperative day 4. However, the levels in group H were lower than those in group C (^#^ *P* < 0.05 versus group C).

The Pp38/p38 levels in group C were significantly increased after the operation (*P* < 0.05 versus day 0) and peaked on postoperative day 4. A similar trend was observed in group H, although the increase was lower than that in group C (*P* < 0.05 versus group C) (Figure 3).

### 3.4. pNF-*κ*B/NF-*κ*B Levels in the Flaps

**Figure 4:**
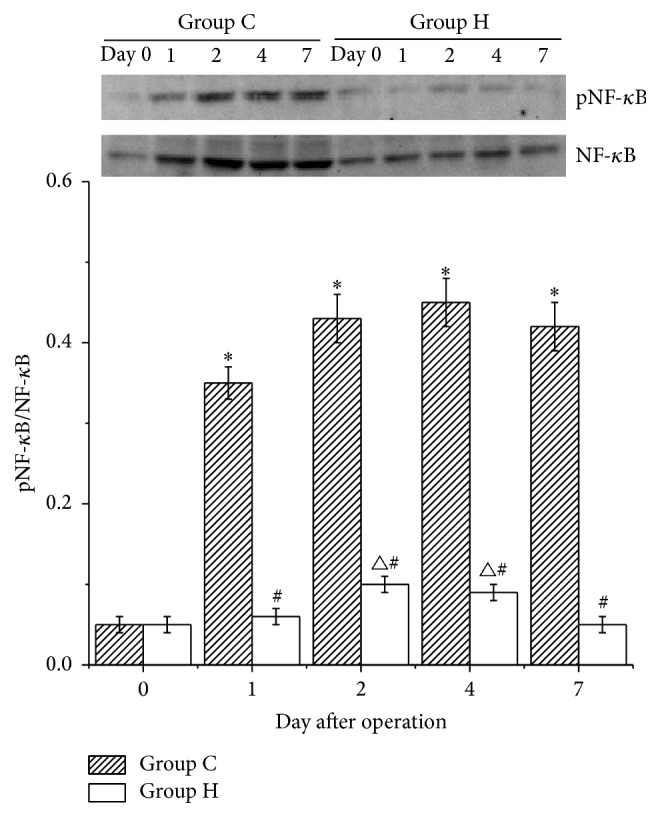
pNF-*κ*B p65/NF-*κ*B p65 levels in groups C and H. The pNF-*κ*B P65/NF-*κ*B p65 levels in group C were significantly increased after the operation (^*∗*^ *P* < 0.05 versus day 0). The pNF-*κ*B p65/NF-*κ*B p65 levels in group H were significantly increased on postoperative days 2 and 4 (^△^ *P* < 0.05 versus day 0). The pNF-*κ*B p65/NF-*κ*B p65 levels in group H were lower than those in group C after surgery (^#^ *P* < 0.05 versus group C).

The pNF-*κ*B p65/NF-*κ*B p65 levels in group C were significantly increased starting on postoperative day 1 (*P* < 0.05 versus day 0). In group H, these levels were increased after the operation and were significantly increased on postoperative days 2 and 4. However, no significant differences were observed between postoperative day 0 and day 7. The pNF-*κ*B p65/NF-*κ*B p65 levels were lower in group H than those in group C after surgery (*P* < 0.05 versus group C) (Figure 4).

## 4. Discussion

Khouri and coworkers advocated a caudally based 3 cm × 10 cm dorsal rat flap and reported that it was the most suitable choice for experimental flap studies [5]. Several authors have used this flap model in their investigations [6–8]. Thus, we also applied the same model in our study and found that the areas of flap necrosis in group C were similar to those in the control groups used in other studies.

The factors that cause flap necrosis include ischaemia, hypoxia, activation of the coagulation system, vascular thrombosis, venous congestion, and inflammation [9–11].

Procedures involving the transfer of random skin flaps require an obligatory period of ischaemia, which can initiate many inflammatory cytokines and produce damaging effects in tissues. In the present study, the significant increase in the TNF-*α*, IL-6, and ICAM-1 levels on postoperative days 2, 4, and 7 in the control group also demonstrated that inflammation was present in the flaps.

Other authors have found that thrombin may cause inflammation in the ischaemic flaps [12]. It has long been known that the main mechanisms responsible for the cellular actions of thrombin are mediated by PARs [13]. Some studies have reported that thrombin can bind to PARs and activate p38-MAPK/NF-*κ*B signalling [14, 15]. Thus, the mechanism underlying the induction of inflammation by thrombin may involve the activation of the PARs/p38-MAPK/NF-*κ*B pathway. The results of this study showed that the p38-MAPK and NF-*κ*B levels in flaps from group C were significantly increased 2 days after surgery, which is consistent with the above hypothesis.

NF-*κ*B is an important transcription factor that can regulate the transcription of a variety of cytokines during inflammation. NF-*κ*B p65 comprises the major portion of the NF-*κ*B dimer. In resting cells, NF-*κ*B/Rel dimers are maintained in the cytoplasm in complex with I*κ*B. Activation of I*κ*B kinase (IKK) can lead to I*κ*B phosphorylation and degradation, which then leads to NF-*κ*B p65 phosphorylation and activation. Activated NF-*κ*B p65 can be transported into the nucleus and combine with *κ*B, which enhances the expression of many cytokines, including TNF-*α*, IL-6, and ICAM-1 [16].

Cytokines involved in the wound healing process play an important role in initiating, controlling, and terminating cellular events such as angiogenesis and extracellular matrix formation [17, 18].

IL-6 and TNF-*α* play key roles in the cross talk between cytokines and are the most readily measurable factors [19, 20]. IL-6 is an important proinflammatory mediator that can activate leukocytes and promote the release of TNF-*α*, which often acts as an indicator of inflammation severity [21].

TNF-*α* is one of the most important proinflammatory cytokines [22]. In surgical wounds, it is secreted into the surgical field first by the stimulation of extravasated and activated monocytes and macrophages. Activated NF-*κ*B, through the PARs/p38-MAPK/NF-*κ*B pathway, also enhances TNF-*α* expression. Increasing TNF-*α* levels can activate IKK, which then degrades I*κ*B and activates additional NF-*κ*B, promoting the further activation of cytokines. ICAM-1 promotes leukocyte accumulation, adhesion to endothelia, transendothelial migration, and the release of tissue-damaging enzymes within the entire flap [23]. The reaction between activated polymorphonuclear neutrophils (PMN) and microvascular vessels is one of the major causes of ischaemic injury [24]. In contrast, leukocyte accumulation causes subsequent microvessel obstruction and oedema formation. Microcirculatory intravascular clotting has been implicated in the pathophysiology of skin flap failure [25, 26].

Natural hirudin is a polypeptide that is isolated from leech saliva extract and is the most active and specific thrombin inhibitor. It is involved in anticoagulation activity, antithrombotic activity, hypolipidemia, changes in viscosity, and anti-inflammatory effects [12, 27].

Many studies have shown that hirudin can increase skin flap viability [6, 28, 29]. The flap viabilities in the present study demonstrated that natural hirudin could significantly increase rat skin flap survival rates (group H versus group C, 88.54 ± 2.81% versus 71.62 ± 3.20%, resp., *P* < 0.05). This is consistent with the results of other authors [29].

The effect of hirudin on increasing flap survival may be related to enhanced angiogenesis and its antioxidation, anticoagulation, and antithrombus activities [6, 28, 29]. However, the exact role of hirudin in anti-inflammatory activity in random pattern skin flaps is still unknown.

In the present study, we found that the levels of TNF-*α*, IL-6, and ICAM-1 in the treatment group were obviously decreased compared to the control group after administration of natural hirudin. This indicated alleviation of inflammation in the treatment group. By measuring the p38 and NF-*κ*B p65 levels, we found that they showed similar trends to those of TNF-*α*, IL-6, and ICAM-1. As hirudin has been shown to compete with thrombin to bind to PAR-1 [27, 30], we deduced that hirudin may attenuate inflammation by inhibiting the interaction of thrombin with PARs. This downregulates p38/NF-*κ*B activation and attenuates thrombin nuclear translocation and thereby DNA binding to RelA/p65.

In summary, the results from this study suggest that the anti-inflammatory functions of the thrombin inhibitor hirudin in ischaemic flaps are likely mediated by mechanisms that involve the downregulation of TNF-*α*, IL-6, and ICAM-1 expression via the PARs/p38-MAPK/NF-*κ*B pathway.

## Abbreviations

PAR: Proteinase-activated receptor
p38-MAPK: P38-mitogen-activated protein kinase
Pp38-MAPK: Phospho-p38-MAPK
NF: Nuclear factor
pNF-*κ*B: Phosphor-nuclear factor-*κ*B
TNF: Tumour necrosis factor
IL: Interleukin
ICAM: Intercellular adhesion molecule
IKK: I*κ*B kinase
PMN: Polymorphonuclear neutrophils
WB: Western blot.
